# Expression of Vesicular Glutamate Transporter 2 (vGluT2) on Large Dense-Core Vesicles within GnRH Neuroterminals of Aging Female Rats

**DOI:** 10.1371/journal.pone.0129633

**Published:** 2015-06-08

**Authors:** Weiling Yin, Zengrong Sun, John M. Mendenhall, Deena M. Walker, Penny D. Riha, Kelsey S. Bezner, Andrea C. Gore

**Affiliations:** 1 Division of Pharmacology and Toxicology, College of Pharmacy, University of Texas at Austin, Austin, Texas, United States of America; 2 Institute for Neuroscience, University of Texas at Austin, Austin, Texas, United States of America; 3 Institute for Cellular and Molecular Biology, University of Texas at Austin, Austin, Texas, United States of America; 4 School of Public Health, Tianjin Medical University, Tianjin, China; John Hopkins University School of Medicine, UNITED STATES

## Abstract

The pulsatile release of GnRH is crucial for normal reproductive physiology across the life cycle, a process that is regulated by hypothalamic neurotransmitters. GnRH terminals co-express the vesicular glutamate transporter 2 (vGluT2) as a marker of a glutamatergic phenotype. The current study sought to elucidate the relationship between glutamate and GnRH nerve terminals in the median eminence—the site of GnRH release into the portal capillary vasculature. We also determined whether this co-expression may change during reproductive senescence, and if steroid hormones, which affect responsiveness of GnRH neurons to glutamate, may alter the co-expression pattern. Female Sprague-Dawley rats were ovariectomized at young adult, middle-aged and old ages (~4, 11, and 22 months, respectively) and treated four weeks later with sequential vehicle + vehicle (VEH + VEH), estradiol + vehicle (E_2_ + VEH), or estradiol + progesterone (E_2_+P_4_). Rats were perfused 24 hours after the second hormone treatment. Confocal microscopy was used to determine colocalization of GnRH and vGluT2 immunofluorescence in the median eminence. Post-embedding immunogold labeling of GnRH and vGluT2, and a serial electron microscopy (EM) technique were used to determine the cellular interaction between GnRH terminals and glutamate signaling. Confocal analysis showed that GnRH and vGluT2 immunofluorescent puncta were extensively colocalized in the median eminence and that their density declined with age but was unaffected by short-term hormone treatment. EM results showed that vGluT2 immunoreactivity was extensively associated with large dense-core vesicles, suggesting a unique glutamatergic signaling pathway in GnRH terminals. Our results provide novel subcellular information about the intimate relationship between GnRH terminals and glutamate in the median eminence.

## Introduction

Reproductive activity is regulated by the coordinated release of GnRH from secretory vesicles in neuroterminals located in the median eminence. The mechanisms by which GnRH terminals release the neuropeptide are complex, as they involve intrinsic processes within the GnRH neurons themselves (e.g., electrophysiological activity) together with the coordination of inputs from other neurotransmitters that may act upon GnRH cells through receptors and intracellular signaling mechanisms. Glutamate, an excitatory neurotransmitter in the hypothalamus, is one such neurotransmitter: it stimulates GnRH gene expression [1, 2], GnRH peptide release [3–5], and GnRH electrical activity [6, 7]. Glutamate is further involved in the reproductive life transitions of puberty [2, 8] and senescence [9–13]. These effects are mediated by glutamate receptors including NMDA and non-NMDA receptors, which are detectable on GnRH cell bodies and terminals [2, 4, 10, 14–16].

Although there is considerable evidence that glutamate is involved in the development and maintenance of adult reproductive function, and that it regulates reproductive senescence, the differential cellular mechanisms of glutamate signaling in GnRH neurons are only just beginning to emerge. Vesicular glutamate transporters (vGluT1, vGluT2 and vGluT3) transport glutamate into secretory vesicles, and are specific markers to identify glutamatergic neurons [17]. Previous studies have shown that vGluT2 mRNA [18] and protein [19] are abundant in the hypothalamus. In relation to the GnRH system, vGluT2 immunoreactive elements are found at high densities in the preoptic region (where GnRH neuron cell bodies are located in rodents), and in the external layer of the median eminence (where GnRH terminals are located) [20, 21]. Electron microscopy evidence showed that vGluT2-immunoreactive boutons made synaptic contacts with GnRH neurons in the medial preoptic area in rats [22] suggesting the importance of glutamatergic regulation of GnRH neuronal function. In addition, several laboratories have suggested that GnRH neurons themselves may be glutamatergic. Hrabovszky *et al*. [21] showed that mRNA of vGluT2, a marker of a glutamate phenotype [17], is highly co-expressed (99%) in GnRH cells in the preoptic region of male rat. Co-expression of vGluT2 GFP signal in at least 84% of GnRH cell bodies was also reported in vGluT2-GFP transgenic mice [23]. In addition, single-cell RT-PCR analysis showed that kisspeptin-activated vGluT2-GFP neurons colocalized with GnRH mRNA in the medial septum/diagonal band [23]. The current study was designed to address, for the first time, the influence of age and hormonal status on vGluT2 density, and vGluT2-GnRH colocalization in GnRH terminal region. We also provided further information on vGluT2 subcellular distribution in the GnRH terminal using a post-embedding electron microscopic approach.

## Materials and Methods

### Animal care

All animal procedures were conducted in accordance with the recommendations in the Guide for the Care and Use of Laboratory Animals of the National Institute of Health. This study was approved by the Institutional Animal Care and Use Committee (IACUC) at the University of Texas at Austin (Protocol Number AUP-2013-00222). Surgery was performed under isofluorane and euthanasia was performed under ketamine/xylazine injection, and all efforts were made to minimize suffering. Female Sprague-Dawley rats were purchased from the Animal Resource Center colony at The University of Texas at Austin. Rats were assigned to use at one of three ages: young adult (4 to 5 months), middle-aged (MA, 11 to 12 months) and old (21 to 24 months). Rats were housed 2 per cage with controlled room temperature (22°C) and light cycles (12 h/12 h light/dark cycle, lights on at 0700 h). General health was checked weekly. Food and water were available *ad libitum*.

### Surgical procedures and hormone treatment

Ovariectomy and hormone capsule implantation procedures were performed as previously described for young, middle-aged, and old groups [16, 24]. Rats of the three ages were bilaterally ovariectomized under isoflurane anesthesia and allowed to recover for 4 weeks to clear endogenous ovarian hormones [25, 26]. Then, Silastic capsules (inner diameter 1.96 mm, outer diameter 3.18 mm; Dow Corning, Midland, MI) filled with either 17-estradiol (5% in cholesterol) or 100% cholesterol (vehicle, VEH) were prepared for young (1 cm in length), middle-aged (1.5 cm in length) and old (2 cm in length) rats [26–28], with differences in capsule length chosen to account for the body weight gain with age. This estradiol treatment regimen has previously been shown by our lab and others to approximate preovulatory estradiol levels at the time of perfusion [26, 29–31]. Capsules were soaked in saline for 24 hours and then subcutaneously implanted into isoflurane-anesthetized rats. After two days (48 hours) of capsule implantation, rats were injected s.c. with either vehicle (0.1 ml sesame oil) or progesterone (0.1 ml, 5.9 mg/ml progesterone in sesame oil) to mimic preovulatory progesterone levels [31, 32]. Thus, there were three hormone treatments for each age: VEH + VEH, E_2_ + VEH, and E_2_ + P_4_, which enable us to evaluate effects of age and different hormone treatment regimens.

### Perfusion and tissue preparation for microscopy studies

Perfusions took place 24 hours after the progesterone or vehicle injection, between 0900 and 1100. Rats were deeply anesthetized with ketamine/xylazine and checked for lack of reflexes. The thoracic cavity was opened, and 3 ml of blood was collected from the left ventricle. Then, rats were transcardially perfused [10, 33, 34]. A cohort of 72 rats was used for the fluorescence microscopic study. The perfusion buffer was PBS (0.122 M PO_4_, 0.077 M NaCl, pH 7.3), and the perfusate sequence was: 0.9% saline (24 ml), 0.9% saline with 10% heparin (24 ml), 1% paraformaldehyde with 3.75% acrolein (48 ml), 4% paraformaldehyde (480 ml). Brains and pituitaries were collected after perfusion and post-fixed for 3 hours in 4% paraformaldehyde, then transferred to PBS [34]. Sections (40 μm thickness) were cut on a vibrating blade microtome (Leica VT1000S, Bannockburn, IL) and stored in PBS with 0.05% sodium azide at 4°C. Antemortem body weight and postmortem pituitary weights were recorded, and the uterine diameter was measured.

Rats for the electron microscopic studies were perfused with 0.1 M phosphate buffer for 1 minute (PB, pH 7.4; 50 ml), followed by 4% paraformaldehyde and 0.125% glutaraldehyde in PB (500 ml) for 10 minutes. The brain was removed and post-fixed in 4% paraformaldehyde overnight at 4°C and then coronal sections (100 μm) were collected on a vibrating blade microtome (Leica VT1000S, Bannockburn, IL). Further tissue processing is described below.

### Fluorescence immunohistochemistry, antibodies, and confocal microscopy

Based on previous observations that GnRH fluorescence intensity is greatest in the caudal median eminence [24], one coronal section per rat was chosen at this level to ascertain whether GnRH terminals co-express vGluT2. Sections were washed in PBS, and blocked in 10% normal goat serum and 10% normal horse serum. Tissues were incubated with rabbit anti-GnRH polyclonal antibody (HU60 at 1:1000, kindly provided by Dr. H.F. Urbanski (S1 Table; [35]) together with the mouse monoclonal anti-vesicular glutamate transporter 2 antibody (vGluT2; 5 μg/ml, MAB5504, Millipore-Chemicon; S1 Table). After overnight primary antibody incubation, tissues were washed in PBS, followed by incubation for 2 hours with secondary antibodies: FITC horse anti-rabbit (1:400, vector laboratory) to detect GnRH, and Texas Red goat anti-mouse (1:400, Vector Laboratories) used to detect vGluT2. The GnRH polyclonal antibody has been in use for two decades and is highly specific to the GnRH decapeptide as determined through radioimmunoassay in Dr. Urbanski’s laboratory [35]. The vGluT2 monoclonal antibody has also been well characterized by several laboratories and its expression has been shown in the hypothalamus and pituitary gland, including the median eminence [21, 36]. Another laboratory used double labeling with this antibody together with another vGluT2 antibody, and showed overlap of the immunoreactive signals [21]. To further verify the specificity of the vGluT2 monoclonal antibody, we performed western blots on protein extracted from the median eminence of young ovariectomized rats. A highly specific band was detected at the expected molecular weight of ~56 kDa.

Confocal microscopy was used to assess the colocalization of GnRH and vGluT2 in the median eminence [24, 37]. From the surface of each tissue section, a stack of 6 images (in a 5 μm thickness with an interval of 1 μm) was captured using a confocal microscope (Leica SP2 AOBS). The fluorescent labeling of GnRH, vGluT2 and colocalization from each stack of images was thresholded at the same level as determined using secondary antibody-only controls that had negligible labeling. The laser parameters were kept constant in a manner to avoid any saturation of immunofluorescence. Immunofluorescent signals were observed as punctate labels along the portal capillary area. For stereologic quantification, a physical disector method [38] was used to quantify the density of GnRH puncta, vGluT2 puncta, and GnRH-vGluT2 co-expressed puncta in the pericapillary area within approximately 40 μm of the portal capillary (S1 Text). This method uses two images, and counts only the objects that were observable in the first section (reference section) but not in the adjacent section to avoid double counts. Four pairs of images from each animal were processed using NIH imaging software Image J 1.45s (http://imagej.nih.gov/ij). In each pair of images, the reference image and adjacent image were 1 μm apart. This distance was chosen because it is approximately half the size of the GnRH terminals, which are about 2 μm in diameter as measured at the electron microscopy level [39]. The images were binarized and the regions of interest (ROI) were chosen using five circles (40 μm in diameter) along the portal capillary border. Images were then created into a stack. Using the Z Project function, particles representing the puncta that appeared in both reference and adjacent images were separated by threshold. Puncta detected as particles from the reference image and project image were analyzed. The density of GnRH or vGluT2 puncta in the pericapillary area was estimated as [(# particles in reference image–# particles in project image) / Volume]. Volume was calculated based on the areas of ROI and the distance between two images. To quantify the density of GnRH and vGluT2 colocalized puncta, images from both channels in the same scanning plane were stacked and Z-projected to create a new image as the colocalized reference image to compare with the adjacent colocalized image (S1 Text). The density of GnRH, vGluT2 and co-expressed puncta were compared for effects of age (young, MA, old) and hormone treatment (VEH + VEH, E_2_ + VEH, E_2_ + P_4_) with SPSS 13.0 software for windows, using two-way ANOVA followed by a Bonferroni post-hoc test. An effect was considered significant at P < 0.05.

We also examined the median eminence for vGluT1 immunoreactivity (AB5905, Millipore-Chemicon, 1:1000 or 1:5000; S1 Table). No vGluT1 immunoreactivity was found at either the light or electron microscopic level in the median eminence; however immunofluorescent labeling was seen clearly in the cortex, suggesting that the failure to detect vGluT1 in the median eminence is due to its absence in this latter region, and consistent with other reports [18, 19, 40]. Although we did not characterize the vGluT1 antibody ourselves, the same antibody has been used in western blots and preabsorption controls showing high specificity [41].

### Electron microscopy tissue preparation and sectioning

To study the subcellular localization of vGluT2 and its relationship with GnRH neuroterminals, we performed serial electron microscopy and immunogold labeling [16, 24, 39]. Because of the highly labor intensive nature of this type of analysis, we randomly chose two young and two old vehicle treated rats for these descriptive studies. Sections were prepared identically to our previously published work [24, 39] and methods are summarized briefly here. Tissues were cryoprotected in increasing concentrations of glycerol. A median eminence fragment of approximately 1 mm^2^ in area and 0.1 mm in thickness was dissected from the lateral median eminence and rapidly plunged into liquid propane (-180°C). Cryofixed tissue was then transferred and immersed into 1.5% uranyl acetate in anhydrous methanol in an Automatic Freeze-Substitution System unit (Leica AFS, Vienna). Following several temperature shifts, the samples were infiltrated with Lowicryl HM20 resin (Electron Microscopy Sciences, Fort Washington, PA), then polymerized under UV light. To process the serial sections, the embedded tissue blocks were trimmed, and a final block surface of approximately 50 μm by 2 mm was chosen to span the pericapillary zone of the lateral median eminence [39]. Sections (50 nm in thickness) were collected in series on formvar-coated gold slot grids (Electron Microscopy Sciences, Fort Washington, PA).

### Immunogold labeling and serial electron microscopy

Post-embedding immunogold labeling was performed as described previously [24, 39]. First we performed double labeling of GnRH and vGluT2 using the GnRH rabbit polyclonal antibody (HU60 at 1:1000 [35]; S1 Table) together with the mouse anti-vGluT2 monoclonal antibody (MAB5504 at 5 μg/ml, Millipore-Chemicon; S1 Table). Sections on slot grids were blocked in 2% human serum albumin and then incubated in primary antibody for 4 hours. After washing in TBS, sections were incubated in secondary antibodies for 1 hour followed by extensive washing. The gold-tagged F(ab’)2 of goat anti-rabbit IgG (5 nm, 1:20, Electron Microscopy Science), gold-tagged F(ab’)2 of goat anti-mouse IgG (10 nm, 1:20, Electron Microscopy Science) were used for GnRH and vGluT2 detection, respectively.

Second, because polyclonal antibodies are not optimal for use in postembedded cryosubstituted tissues due to high background, we used adjacent ultrathin sections for single labeling with a GnRH monoclonal antibody (HU11b at 1:100, kindly provided by Dr. H.F. Urbanski [16, 42]; S1 Table) on one section, and the vGluT2 monoclonal antibody (MAB5504) on the adjacent section. The same nerve terminals can be identified from section to section; thus, if the GnRH antibody labels the terminal in the first section and the vGluT2 antibody labels the same terminal in the next section, this can confirm co-expression of the two antigens. The GnRH HU11b antibody has been used in our previous electron microscopy papers [24, 39], and preabsorption control experiments eliminated specific labeling [16, 42]. Sequential images were taken using a transmission electron microscope (Philips EM208, Eindhoven, Netherlands) with a digital camera (AMT HR 1Mb).

To further evaluate the localization of vGluT2 immunoreactivity, a representative immunopositive neuroterminal in the median eminence was reconstructed using the serial TEM 3D reconstruction technique described previously [39]. Sequential images of the vGluT2-immunopositive neuroterminal from section to section were aligned using software Reconstruct [43](www.synapse-web.org/tools/index.stm). The immunopositive nerve terminal, surrounding glial elements and portal capillary were outlined. The distribution of vGluT2 gold bead labels, vesicles and mitochondria were carefully traced and reconstructed.

### Radioimmunoassay (RIA) for estradiol and progesterone

Serum samples collected at the time of perfusion were centrifuged at 4000 rpm for 8 min, aliquoted and stored in a– 80°C freezer for subsequent estradiol and progesterone assays. Serum estradiol concentrations were determined by RIA of duplicate samples using the DSL ultrasensitive estradiol RIA kit (DSL-4800). This assay sensitivity was 2.2 pg/ml, and intra-assay variability was 6.1%. Serum progesterone concentrations were determined in a coated tube RIA using the DSL progesterone kit (DSL-3900, Diagnostic Systems laboratories, Inc., Webster, TX) following the manufacturer’s instructions. Samples were run in duplicate in a single assay. The sensitivity of this assay was 0.12 ng/ml, and intra-assay variability was 6.6%. Statistical analyses comparing effects of age and hormone treatment, and their interactions were performed by two-way ANOVA followed by Bonferroni post-hoc test using SPSS 13.0 for windows. Statistical significance was set at *P* < 0.05.

## Results

### GnRH and vGluT2 immunofluorescence puncta in the lateral median eminence

**Fig 1:**
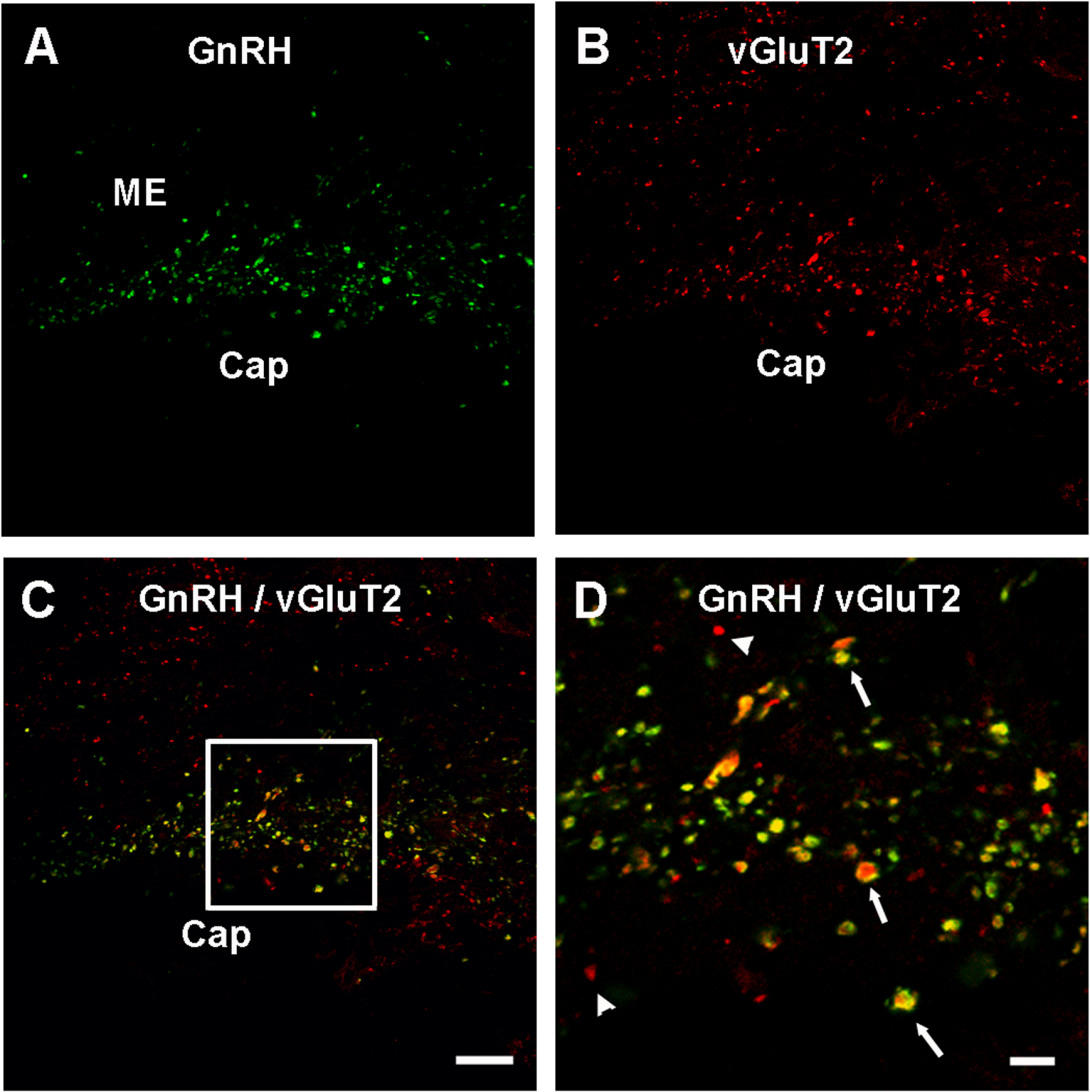
Confocal microscopic images show GnRH and vGluT2 co-localization in the lateral median eminence (ME). Images were scanned from a single plane of a representative middle-aged vehicle treated rat. (A) GnRH processes were labeled with FITC (green color) and are seen along the portal capillary region (Cap) in the caudal median eminence. (B) Texas Red signals (red color) representing vGluT2 were clearly seen in the pericapillary area with a similar pattern to that of GnRH. (C) A merged image showed colocalization of GnRH and vGluT2 fluorescent signal. (D) A region from panel C (framed) is shown at higher magnification. Some punctate structures were single labeled by vGluT2 (arrow head); however, considerable overlapping of GnRH and vGluT2 signals were seen in yellow (arrows). Scale bar (shown in panel C, applies to panels A-C) = 20 μm, scale bar D = 5 μm. Cap = portal capillaries,

**Fig 2:**
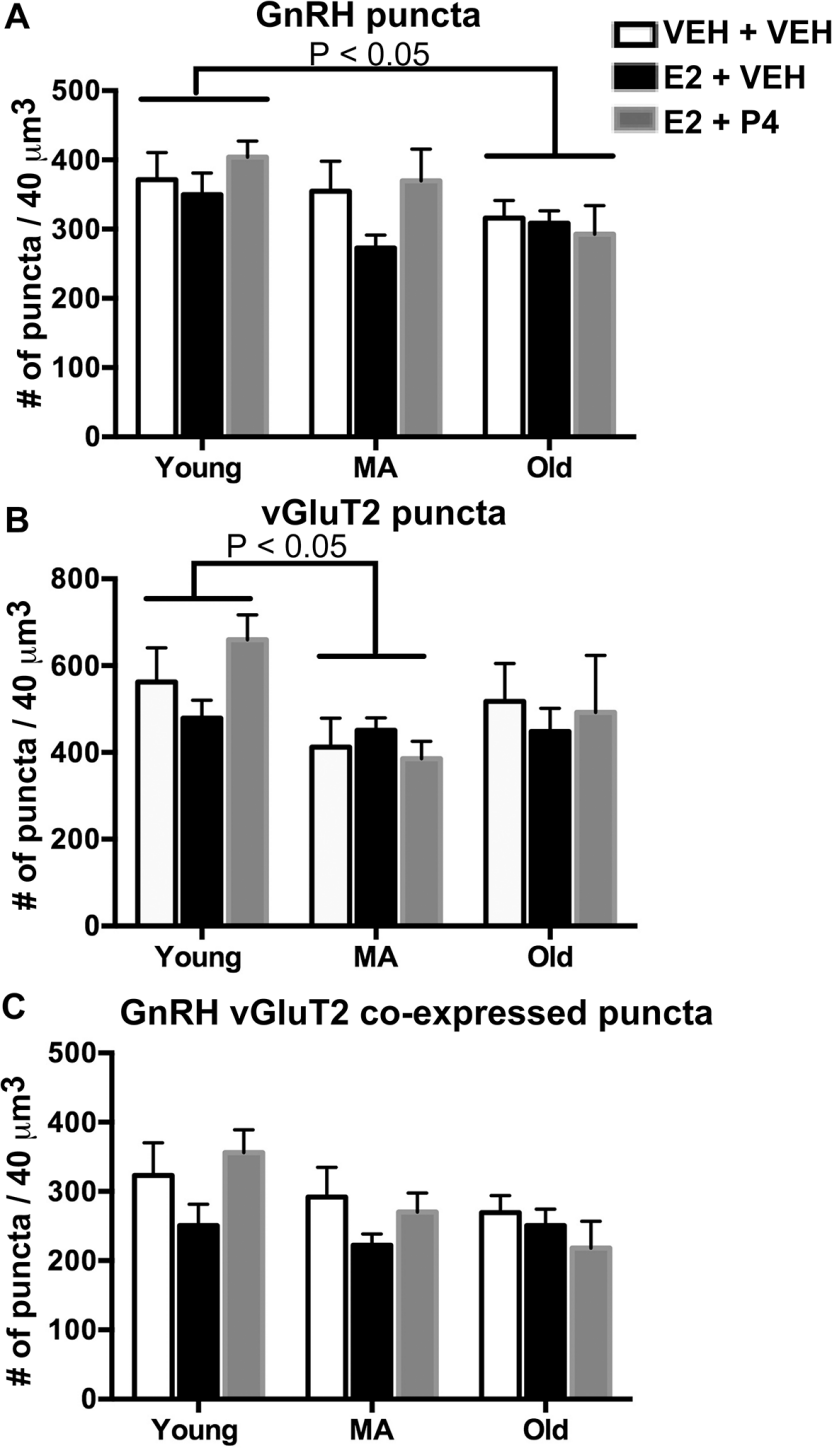
Quantification of the density of GnRH, vGluT2 and co-expressed puncta in the pericapillary area of the median eminence of young, middle-aged, and old rats, treated with vehicle or hormones. (A) The number of GnRH puncta per 40 μm^3^ were calculated and showed a decrease with age from the young to the old group (P < 0.05). (B) The density of vGluT2 puncta decreased with age from the young to the MA group (P < 0.05). (C) GnRH-vGluT2 colocalized puncta did not change significantly with age although a trend was found (P = 0.054). N = 8 rats per group.

In the lateral ME, GnRH and vGluT2 immunofluorescence was detectable under the confocal microscope as punctate labeling (Fig 1), indicative of clusters of immunopositive vesicles observed at the electron microscopy level. Confocal microscopy showed that the vGluT2 puncta considerably overlapped with GnRH puncta (Fig 1). The density of GnRH puncta showed a significant main effect of age (F = 3.306, P < 0.05) (Fig 2A), and the post-hoc test showed a significant decline from young to old. There was no significant effect of hormone (P = 0.22) and no interaction of age and hormone (P = 0.50). The density of vGluT2 puncta (Fig 2B) also showed a significant main effect of age (F = 3.333, P < 0.05), with a significant decline between the young and MA rats. No significant effects of hormone (P = 0.64), or an interaction of age by hormone (P = 0.52), were found.

The density of colocalized GnRH and vGluT2 puncta was quantified in the lateral ME (Fig2C). Although there was extensive co-expression of the signals, we only detected a non-significant trend (F = 3.057, P = 0.054) for an age-related decline. No significant effects of hormone (P = 0.34), or interaction of age and hormone (P = 0.13), were found.

### Co-expression of vGluT2 in GnRH terminals by electron microscopy

**Fig 3:**
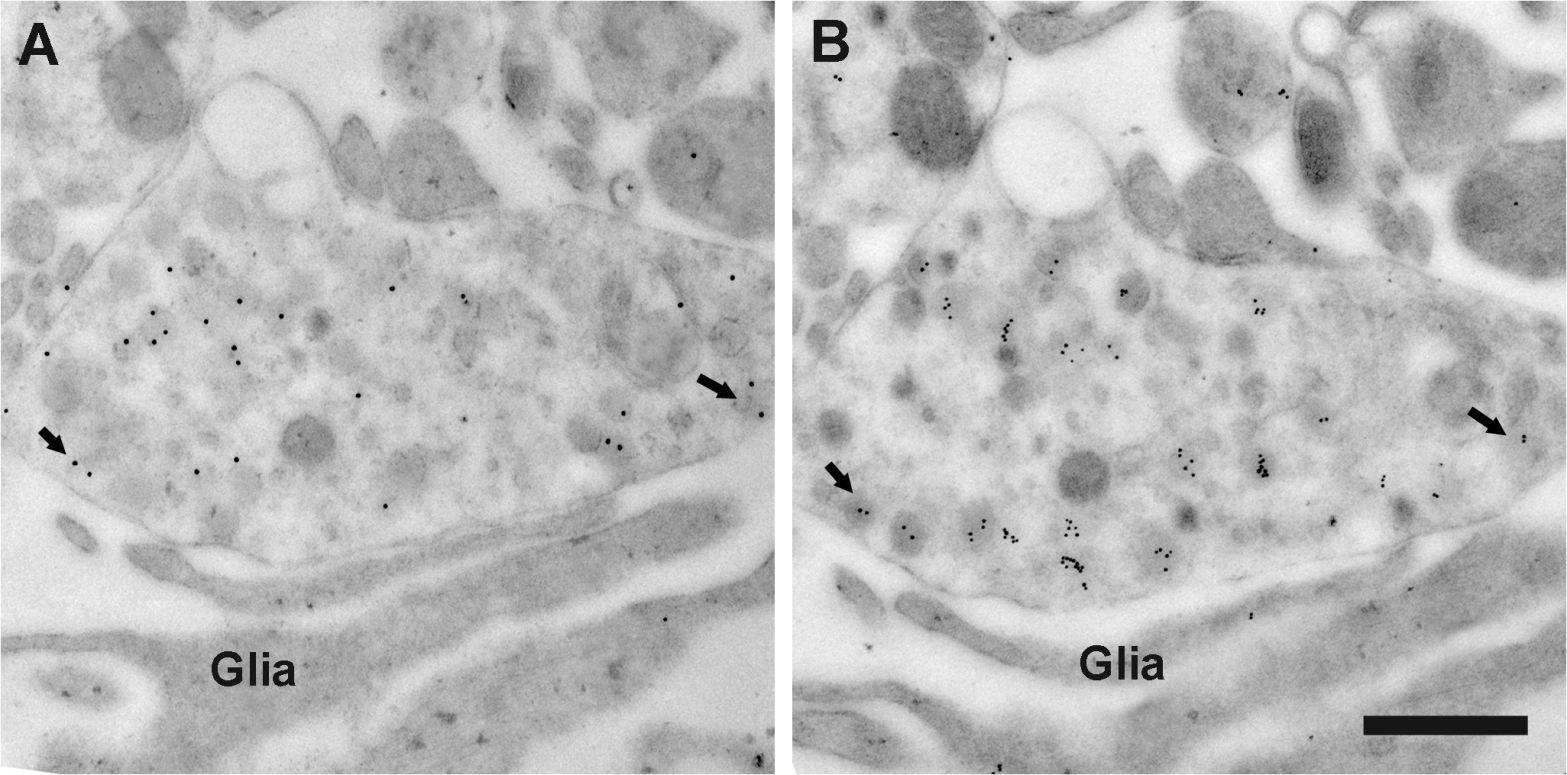
Electron micrographs show GnRH and vGluT2 immunogold labeling on adjacent sections. Immunogold labeling was performed on two adjacent ultrathin sections from a representative young vehicle treated female rat. (A) A GnRH immunogold labeled terminal (15 nm gold particles) was shown. (B) The adjacent section was used for vGluT2 single label with 10 nm immunogold. In both images, immunogold labels were associated with large dense-core vesicles. The same large dense-core vesicles were seen containing both GnRH and vGluT2 immunogold labels (as indicated by arrows) in the adjacent sections. Glial processes (Glia) were in close contact with this GnRH / vGluT2 immunoreactive terminal. Scale bar = 500 nm.

**Fig 4:**
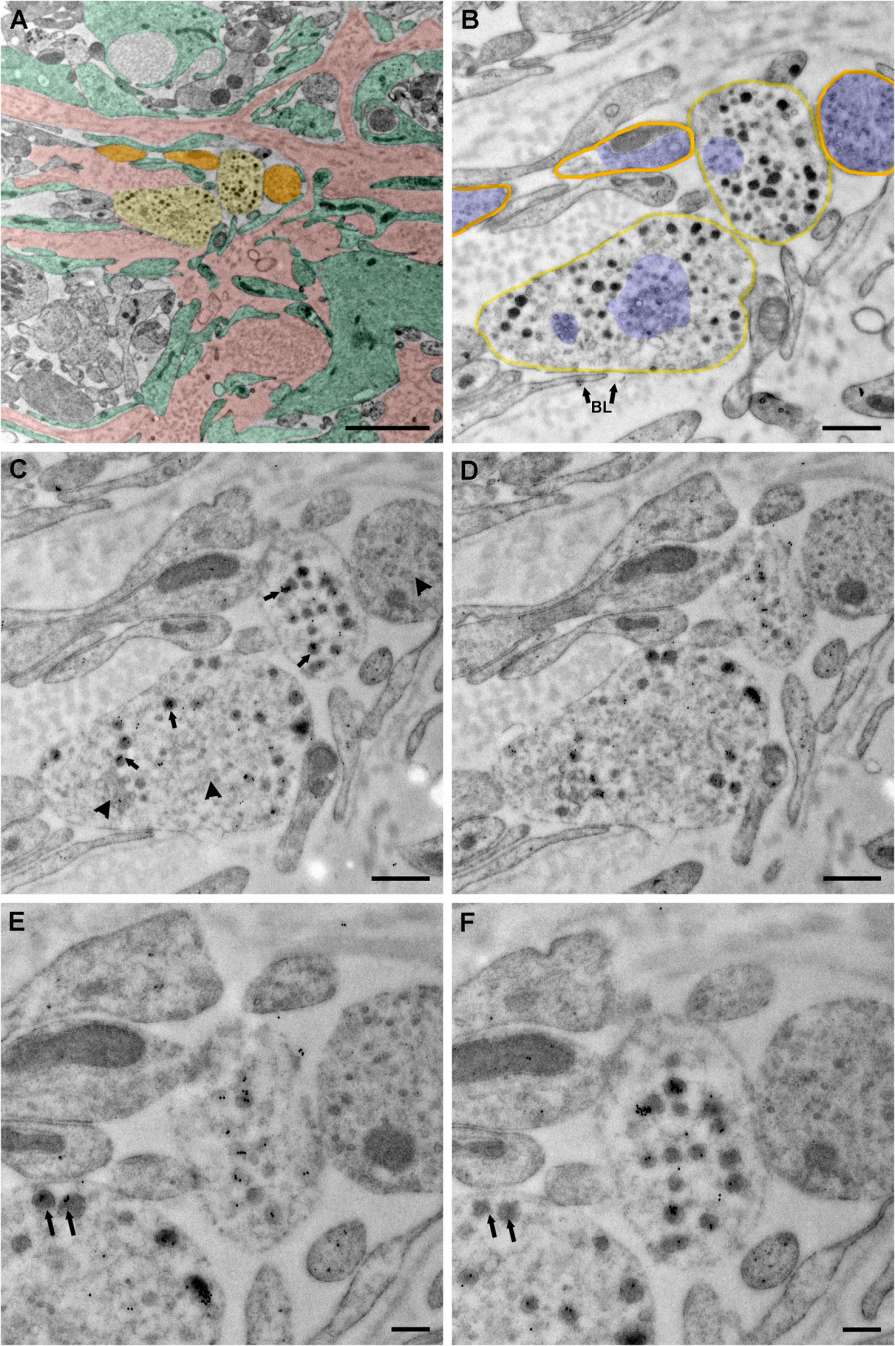
Serial sections show subcellular distribution of vGluT2. Images were taken from a representative middle-aged vehicle treated female rat. (A) A low magnification electron microscopy image was pseudocolored to show neuroterminals with (light yellow) and without (orange) large dense-core vesicles, a convoluted basal lamina covered portal capillary area—typical in older rats (pink), and tanycytic elements (green). (B) A higher magnification image of panel A was shown. This section was used as a negative control, in which omitting primary vGluT2 antibody resulted in no immunogold labels in the terminal. Two neurosecretory terminals containing large dense-core vesicles in direct contact with the basal lamina (BL with arrow) of the portal capillary region were highlighted in light yellow, and surrounding neuronal elements without large secretory vesicles were highlighted in orange. Groups of small vesicles in neurosecretory terminals and surrounding neuronal elements were pseudocolored in blue. A cluster of small vesicles was distributed in the central portion of the lower left terminal. Another cluster of irregular shaped small vesicles was shown to the left. (C, D) Serial sections adjacent to panel B were used to label vGluT2 protein. Immunogold labels for vGluT2 were seen on large dense-core vesicles (arrow). Whether small vesicles (arrowhead) contained vGluT2 immunosignal was difficult to determine, even at higher magnification. (E, F) By examining adjacent sections, it could be seen that immunogold labels of vGluT2 were present in most dense-core vesicles. As indicated by double arrows, some large dense-core vesicles in image E showed no immunogold labels, while the same vesicle in the adjacent image F was immunolabeled. This is likely to be the case because the antibody reaction site on large dense-core vesicle (about 120 nm diameter) might only be captured in sections (each about 50 nm thick) in which antigen is exposed. BL (panel B) = basal lamina. Scale bar panel A = 2 μm, B-D = 500 nm, E-F = 200 nm.

**Fig 5:**
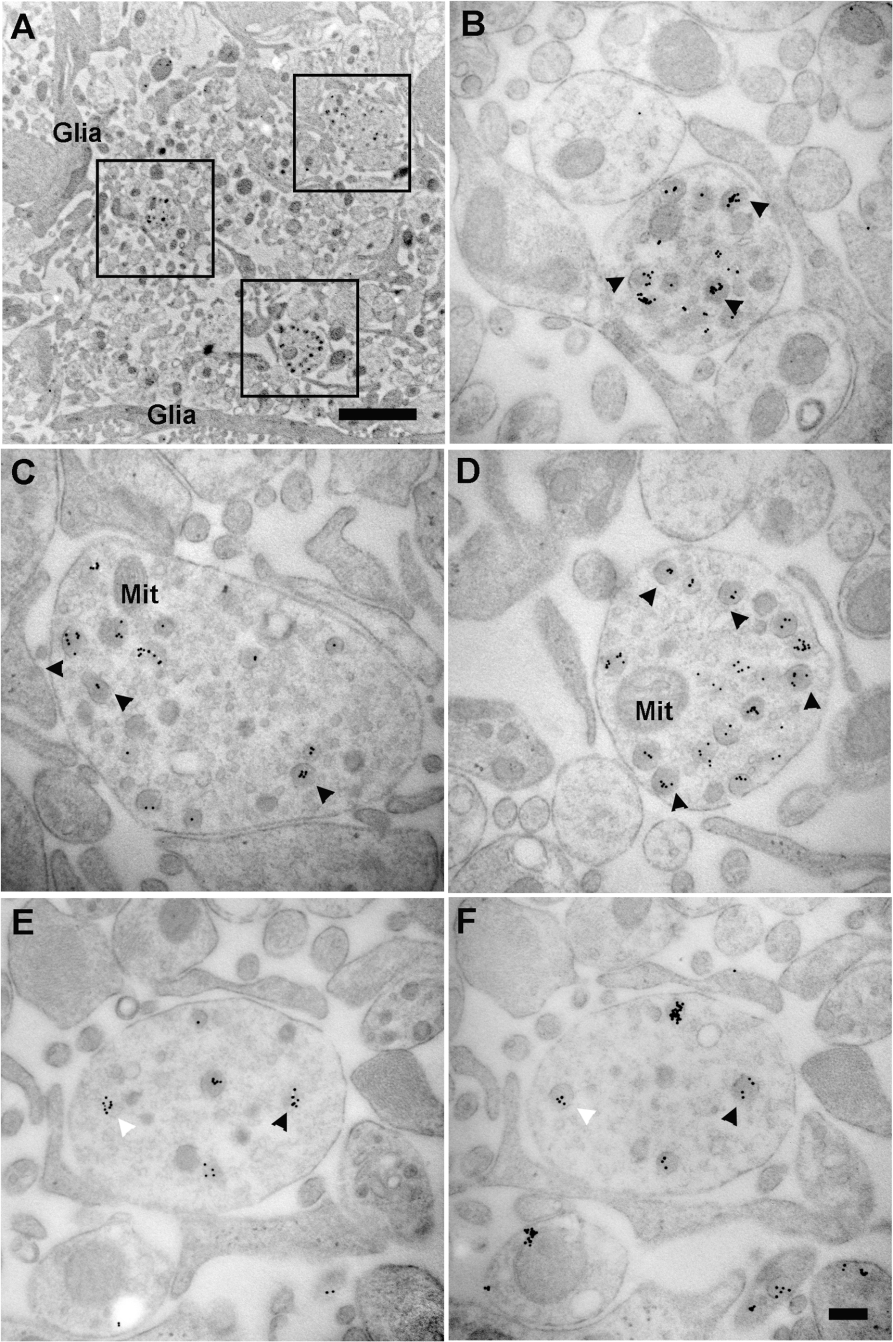
Electron microscopy images show the subcellular distribution of immunoreactive vGluT2 in neural profile of a representative young VEH treated rat. Images were taken from the neuroprofile zone of the lateral median eminence, a region in which neurosecretory axons and GnRH dendrons are located. (A) Three puncta were framed and shown in higher magnification in panels B, C, and D. (B—D) The 10 nm immunogold labels for vGluT2 were seen on large dense-core vesicles (arrowheads). (E, F) Two representative adjacent sections from a series show consistent immunogold labeling of vGluT2 on large dense-core vesicles (white and black arrowheads). Glia = glial processes, Mit = mitochondria. Scale bar panel A = 2 μm, panel F (applies to B–F) = 200 nm.

**Fig 6:**
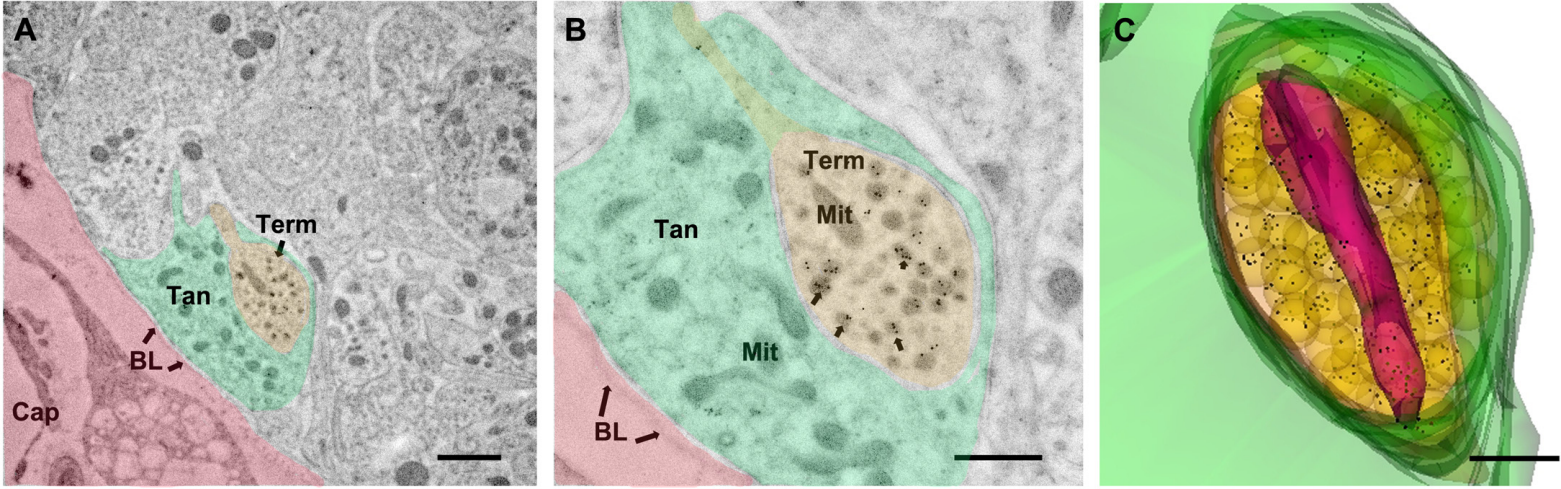
3D reconstruction of electron microscopy image showed immunogold labels of vGluT2 associated with large dense-core vesicles in a neuroterminal of a representative middle-aged VEH treated rat. (A) Lower magnification electron microscopy image was taken from the portal capillary zone of the lateral median eminence. A vGluT2 immunolabeled neuroterminal (Term, pseudocolored in light orange) was separated from the portal capillary area (pseudocolored in pink) by tanycyte endfeet (Tan, pseudocolored in green). (B) An adjacent section showed the same vGluT2 immunopositive neuroterminal from panel A in higher magnification. The 10 nm immunogold labels for vGluT2 were seen on large dense-core vesicles (arrows). (C) The terminal shown in panel A and B was 3D reconstructed from 13 images taken from a serial section ribbon. Reconstruction showed this terminal was covered by tanycyte endfeet (green). The majority of the vesicles in this terminal were large dense-core vesicles (yellow). Immunogold beads labeled for vGluT2 were individually marked in black dots and showed extensive association to large dense-core vesicles. A large mitochondria (purple) was located in the center of the terminal. BL = basal lamina, Mit = mitochondria, Tan = Tanycyte, Term = Neuroterminal. Scale bar panel A = 100 μm, B = 500 nm, C = 200 nm.

In order to confirm whether GnRH neurons co-express vGluT2, we used the immunogold labeling technique for double labeling of ultrathin sections of the lateral median eminence, as well as single labeling of adjacent serial sections by GnRH and vGluT2 monoclonal antibodies. Double labeling experiments showed that GnRH and vGluT2 immunoreactivity were co-expressed in the same terminals, but the polyclonal GnRH antibody labeling had high background (data not shown). Therefore, we turned to adjacent serial sections to perform immunocytochemistry for GnRH and vGluT2 using their respective monoclonal antibodies. This method resulted in very specific labeling in a subset of terminals in the median eminence, and enabled us to show that the same terminal in adjacent sections could co-express GnRH and vGluT2. Vesicles of different sizes (30 nm to 140 nm in diameter) were found in GnRH terminals. Interestingly, the majority of vGluT2 seemed to be associated with large dense-core vesicles in neuroendocrine terminals **(**Figs 3 and 4) and processes (Fig 5) in the lateral median eminence. In serially sectioned preparations, immunogold labels for vGluT2 were on most large dense-core vesicles. Even if labeling was not seen on a dense-core vesicle in one section, the same vesicle in the adjacent section was often labeled (Figs 4 and 5). This is because the diameter of large dense-core vesicles is about 100 to 140 nm, while individual sections were only about 50 nm thick with a single section face exposed to the antibody during incubations. Therefore, the antibody reaction site on large dense-core vesicle might only be captured on section faces in which the antigen site is exposed (Figs 4 and 5). Three-dimensional reconstruction of terminals mainly containing large dense-core vesicles also showed that almost all of the large dense-core vesicles in GnRH terminals were vGluT2 immunopositive (Fig 6). Although quantitative analyses were not performed, there were no apparent differences in vGluT2 distribution in young (Fig 3) and old (Fig 4) vehicle treated rats.

### Serum hormones and characteristics of rats

**Fig 7:**
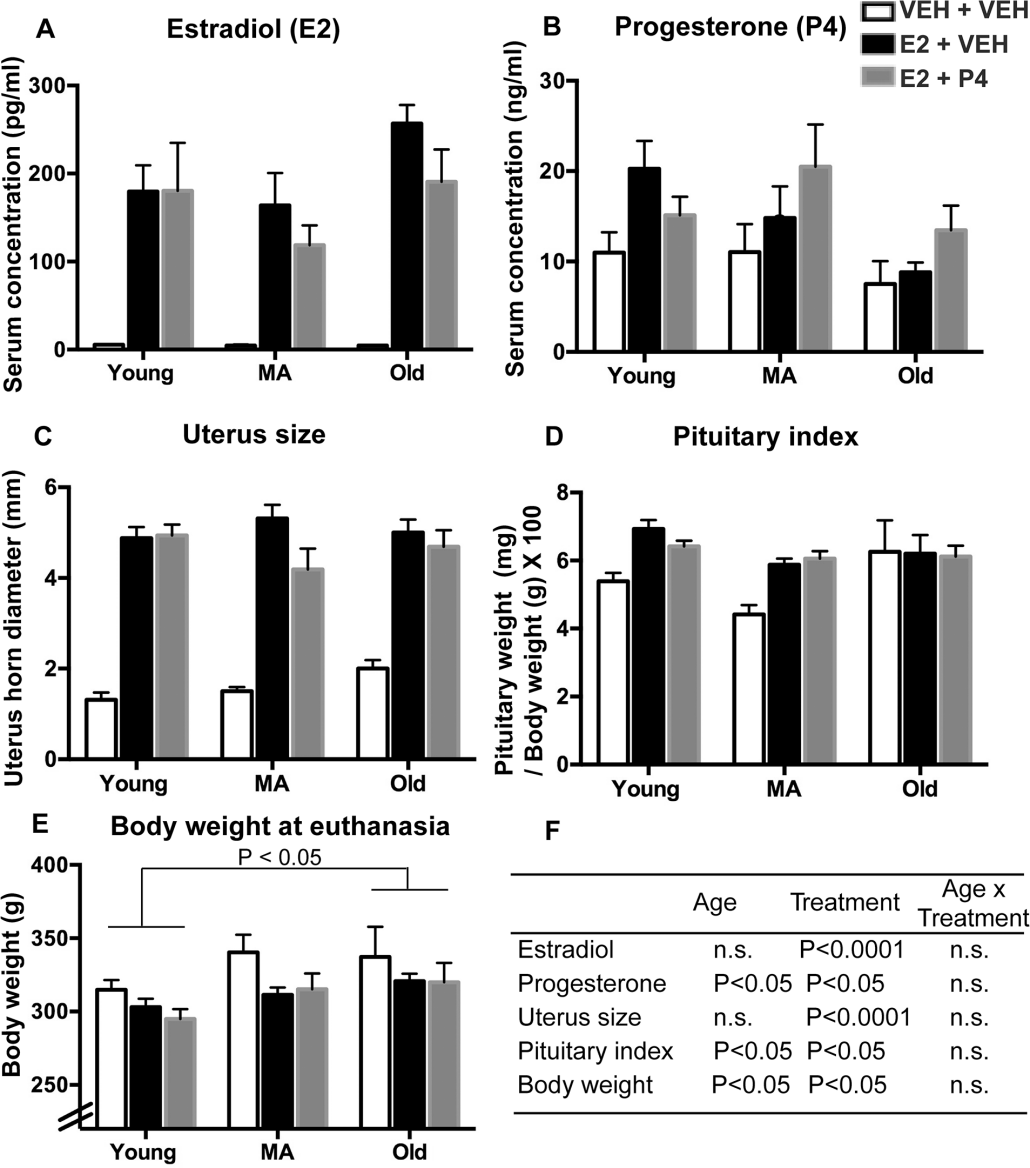
Serum hormones and physiological parameters were measured to evaluate treatment outcomes. Serum estradiol levels were assayed in a subset of rats in each group (N = 3–7 per group). Other parameters were measured in all rats (N = 8 per group). (A) Serum estradiol levels at all ages were significantly elevated by E_2_ in both E_2_ + VEH (P < 0.0001) and E_2_ + P_4_ (P < 0.0001) groups, compared to VEH + VEH treatment. (B) Serum progesterone concentrations were significantly altered by hormone treatment (F = 3.91, P < 0.05) with the E_2_ + P_4_ group higher than VEH + VEH group. (C) The diameter of the uterine horn was significantly altered by hormone treatment (F = 114.9, P < 0.0001) with both E_2_ +VEH (P < 0.001) and E_2_ + P_4_ (P < 0.001) higher than VEH + VEH group. (D) Pituitary weight index showed a main effect of age (F = 3.47, P < 0.05) and hormone (F = 4.67, P < 0.05) with E_2_ +VEH higher than VEH + VEH group. (E) Body weight was significantly altered by age (F = 4.06, P < 0.05), with the young group lighter than old group (P < 0.05). There was a significant treatment effect on body weight (F = 3.235, P < 0.05), with significantly lower weight in the E_2_ + P_4_ group (p < 0.05), and a trend for lower weight in the E_2_ + VEH group (p = 0.08) compared to VEH + VEH control group. (F) Statistical results were summarized to show main effects of age, treatment, and age x treatment interactions. n.s. = not significant. Abbreviations: MA: middle-aged, E_2_: estradiol; P_4_: progesterone; VEH: vehicle treatment.

Serum estradiol and progesterone concentrations, uterus horn diameter, pituitary index and body weight at euthanasia were used to evaluate age and hormone treatment effects. Results and detailed statistics are shown in Fig 7. As expected, 3 days of E_2_ treatment significantly elevated serum estradiol in both E_2_ + VEH and E_2_ + P_4_ groups compared to VEH groups (P < 0.0001). There were no differences in serum E_2_ concentrations between age groups. Serum progesterone was higher in the E_2_ + P_4_ group compared to VEH treated groups (P < 0.05). Changes of physiological parameters in response to 3 days of hormone treatment were also seen. Body weight, diameter of uterus horn and pituitary weight index were all significantly altered by hormone treatment (Fig 7).

## Discussion

The pulsatile release of GnRH is crucial for maintaining reproductive function, and this pattern is altered during reproductive aging in a species-specific manner. Previously, using an aging rat model, we showed a number of ultrastructural changes in GnRH terminals, and their relationship to glia in the median eminence, that may be related to functional changes in GnRH release [24, 39]. Here we further tested the potential role of glutamate, and its relationship to GnRH terminals in the median eminence. We applied this question to aging female rats, and in addition, we investigated hormonal regulation of this phenomenon.

### Co-expression of vGluT2 in GnRH terminals in the median eminence

The median eminence is a very unusual brain structure, as it contains nerve processes but not perikarya or dendrites; it has few if any synapses; and it likely communicates by volume transmission as opposed to synaptic transmission (reviewed in [44]). Evidence on the regulation of GnRH release from neuroterminals implicates the glutamatergic system as playing an important role. Glutamate receptors are present in the median eminence, as shown by immunohistochemistry [4, 15, 16]. Perifusion and push-pull perfusion studies of this region also show that glutamate is released [45, 46]. Recent morphological and functional study of GnRH identified that the GnRH “dendron”, which has a mixed phenotype of dendrites and axons, is responsive to glutamate stimulation within and near the median eminence [47]. Furthermore, vGluT2 is expressed in the median eminence, including the external zone in proximity to the portal capillary vasculature [20, 21, 40, 48]. Therefore, there is consensus that glutamatergic transmission in this region plays a role in mediating neuroterminal function.

Interestingly, some limited evidence also suggests that a GnRH neuron itself could be glutamatergic. A study using *in situ* hybridization showed that 99.5% of GnRH perikarya in preoptic area co-expressed vGluT2 mRNA in adult male rats [21]. GnRH terminals in the median eminence are also immunopositive for vGluT2 [21]. Another study used vGluT2-GFP mice to report that at least 84% of GnRH cell bodies co-expressed vGluT2 [23], a result that the authors speculated may be an underestimate. Our current finding confirms and extends these findings with an approach that adds in additional subcellular compartmental specificity. By using post-embedding immunogold electron microscopy on serial ultrathin sections and 3D reconstruction technique, we showed that GnRH secretory vesicles co-express the vGluT2 protein.

At the electron microscopy level, vGluT2 immunogold labels were easily seen in a subset of terminals that contained large dense-core vesicles in the median eminence. At the light microscopy level, vGluT2 fluorescent signals showed a punctate appearance and were localized both on GnRH terminals and scattered in the median eminence. Hrabovszky *et al*. demonstrated that many types of hypothalamic neuroendocrine cells (GnRH, thyrotropin-releasing hormone, corticotropin-releasing hormone, somatostatin, oxytocin, vasopressin) contain vGluT2 mRNA, and the terminals of those neurons in the median eminence contain vGluT2 protein immunolabels [49–51]. This evidence suggests that a unique internal glutamatergic signaling pathway may be involved in the neural hormone transmission in the hypothalamus. We do not know whether and how the internal vGluT2 related glutamate signals interact with the external glutamate synaptic/volume transmission surrounding GnRH cell bodies and terminals. Together with the evidence that vGluT2 immunopositive boutons are in close proximity to GnRH cell bodies based on a light [20] and electron microscopy [22] study, we suspect that both internal and external glutamatergic signaling are important for GnRH neuronal function.

While vGluT1 immunoreactivity was detectable in the preoptic area and hypothalamus, it was never in proximity to GnRH neurons [22]. This result is consistent with our finding of a lack of overlap of vGluT1 and GnRH immunoreactivity in the median eminence, and with other studies showing that vGluT1 labeling is relatively low in the median eminence and hypothalamus compared to vGluT2 labeling [40]. In fact, Fig 1H and 1H’ of Kaneko *et al* [40] show clear labeling of the external median eminence with vGluT2 but the apparent absence of vGluT1. Therefore, vGluT2 appears to be the primary or sole player in the glutamate cycle in the median eminence.

### GnRH and vGluT2 immunofluorescence signals during reproductive aging

We previously reported that GnRH immunofluorescence intensity in the median eminence underwent little change with age among young, middle-aged, and old OVX rats, and further, that there was no difference between 3 days of vehicle and estradiol treatment given a month post-ovariectomy [24]. In the current study, we used a more highly quantitative stereology method to measure the density of GnRH immunopositive puncta along the pericapillary region. We found small but significant age related changes with higher GnRH puncta density in young compared to old rats. There were no differences between vehicle and E_2_ treatment, as shown previously, and our current study add new data showing that there was also no effect of P_4_ on GnRH puncta density in the caudal median eminence. At the light microscopy level, the density of puncta represents the GnRH dendron branches and terminals in close proximity to the portal capillary system [47]. Our results suggest that GnRH dendrons may have fewer branches, and/or fewer GnRH dendrons are present in old rats compared to young. These changes are relatively small, and thus the storage of GnRH in the median eminence is relatively stable across life and is not highly dependent upon steroid hormones in the circulation, at least in the OVX plus short-term hormone treatment model used here. The fact that GnRH release declines with age in rats implies that there may be a deficiency in stimulation of GnRH neurons as opposed to their absolute ability to synthesize and store the peptide.

Consistent with previous reports in male rats [21, 48], we showed a clear pattern of vGluT2 immunoreactivity in the median eminence and considerable overlap with GnRH axons and terminals in female rats. We detected significantly lower vGluT2 puncta density in the lateral median eminence in middle-aged compared to young rats. This decline is consistent with reports from Neal-Perry showing that glutamate release measured by microdialysis in the preoptic area declines with aging [11]. Although that study was conducted in a different part of the hypothalamus, it is indicative of a loss of glutamatergic input to GnRH cell bodies in the aging preoptic area, together with our current finding of a diminution of vGluT2 as a marker for glutamate in the median eminence. However, the colocalization of GnRH and vGluT2 punta density only showed a trend for a decrease (P = 0.054) that, together with the different ages at which GnRH (young to old) and vGluT2 (young to MA) decreased, suggests that the age-related decline of vGluT2 in the median eminence was probably not associated specifically with the GnRH system. Functionally, it is possible that the overall decline in vGluT2 signal in the median eminence with aging may contribute to a lack of stimulation of pulsatile GnRH release [52–54]. Even though we failed to detect a short-term estradiol effect on vGluT2 puncta density in the median eminence, recent work in our laboratory using long-term estradiol treatment (3 months of hormone treatment duration after OVX) showed significantly lower expression of the vGluT2 gene (*Slc17a6*) in the arcuate nucleus in young but not middle-aged rats (unpublished data). In that same study, vGluT2 gene expression in the medial preoptic area was not altered by age or hormone treatment, suggesting region-specific differences in glutamate regulation in the hypothalamus.

### Expression of vGluT2 on large dense-core vesicles of GnRH immunopositive terminals

Using a serial electron microscopy and multi-probe immunogold labeling technique, we made the surprising finding that a majority of vGluT2 immunolabel was associated with large dense-core vesicles also containing GnRH peptide. We cannot draw any conclusions about the association of vGluT2 on small vesicles [48] as the small and large vesicles are often intermingled, and serial cutting of 50 nm thickness sections does not enable identification of the same small vesicle (normally < 40 nm in diameter) on adjacent sections. Inspection of terminals mainly containing large dense-core vesicles confirmed that vGluT2 immunogold labels were associated with large secretory vesicles. The high resolution of post-embedding immunogold work shown herein and in other studies [16, 24, 39] enables us to distinguish the subcellular compartmentalization and supports labeling of large dense-core vesicles with vGluT2 in GnRH terminals.

The novel subcellular location of vGluT2 in GnRH neuroterminals suggests that GnRH neurons themselves can synthesize and potentially co-release glutamate with the GnRH decapeptide. To our knowledge, this is the first evidence of potential co-release of a neurohormone and glutamate from same large dense-core vesicles in the central nervous system. Interestingly, in the peripheral endocrine system, vGluT2 is expressed in secretory granules of glucagon-secreting alpha cells in the pancreas [55]. Co-secretion of these secretory contents (glutamate and glucagon) from pancreatic alpha cells is trigged by low glucose conditions and further triggers gamma-aminobutyric acid (GABA) secretion from beta cells [55]. In the hypothalamus, the collaborative function between GABA and glutamate also plays an important role in regulating reproductive function [47, 56, 57]. A pattern of an increase in glutamatergic and decrease in GABAergic synaptic inputs to GnRH neurons at proestrus were shown in young [57], but not in middle-aged rats [58] and mice [59]. There are also robust effects of GABA in the regulation of GnRH secretion at the median eminence [60, 61]. The interaction between glutamate and GABA signaling pathway [61, 62], and relationships to kisspeptin signaling (a potent neuropeptide activator of GnRH release) indicates the complexity and region specific regulation of GnRH release [63–65]. Although recent studies show that kisspeptin nerve elements make direct contacts on GnRH terminals without synaptic structures in the median eminence [66, 67], and that kisspeptin directly regulates GnRH release from the median eminence [65], whether and how the glutamate signaling is involved is still unclear and requires future study. It has been suggested that GnRH neuroterminals and their surrounding modulatory inputs including kisspeptin and glutamate/GABA forming a signaling network that regulates GnRH release in the median eminence [47, 65]. Our microscopic evidence of the potential for co-release of glutamate and GnRH suggests a non-synaptic glutamate signaling pathway.

Considering that the median eminence is sparse in synapses, we believe that this region may have co-opted receptor-ligand regulated mechanisms for alternative functions within and between nerve terminals. It is noteworthy that specialized glial cells such as tanycytic are actively involved in communication of GnRH processes and terminals with the surrounding microenvironment [24, 39, 68–71]. The interactions between tanycytes and GnRH neuron processes are dynamic, with changes with hormone status [71–73] and age [24, 39, 68, 74]. The signaling pathways responsible for this plasticity involve many diffusible gaseous molecules, paracrine communications and soluble proteins acting through receptors in specialized glial-neuronal junctions [70, 71]. We believe that tanycyte also actively involved in the non-synaptic glutamate network. Tanycytes show strong expression of glutamate transporters [75] suggesting a role of tanycyte-mediated glutamate transport. It is possible that intracellular glutamate may enable calcium transport and participate in the release of GnRH. After GnRH and glutamate co-release from large dense-core vesicles, tanyctes regulate glutamate re-uptake and recycle. Clearly, much more research is needed to test this hypothesis, and to differentiate these functions from the traditional role of glutamate in extracellular signaling via the glutamate receptor.

## Conclusions

Our findings provide further evidence that glutamate plays an important role in GnRH release. To our knowledge, the large dense-core vesicle localization of glutamate transporters in the central nervous system has not been shown before and therefore, its functional importance is unknown and need further investigation. We speculate that vGluT2 transports and packages glutamate within the GnRH neural process and that glutamate in large dense-core vesicles may play a role in regulating GnRH terminal function instead of synaptic transmission. Our results suggest a potentially different role of glutamate in stimulating neuroendocrine terminals in the median eminence.

## Supporting Information

S1 Table Primary antibodies. (DOCX)

S1 Text Using confocal images and physical disector method to quantify the density of GnRH and vGluT2 puncta in the median eminence of the hypothalamus. (DOCX)
