# Supplementary material for: Expression of Vesicular Glutamate Transporter 2 (vGluT2) on Large Dense-Core Vesicles within GnRH Neuroterminals of Aging Female Rats
Source: PLoS One. 2015 Jun 8;10(6):e0129633. doi: 10.1371/journal.pone.0129633 (PMC4459826; doi:10.1371/journal.pone.0129633)
Supplement: S1 Table — (DOCX) [file pone.0129633.s001.docx]

**Supplemental Table S1. Primary antibodies**

| Antigen | Immunogen | Manufacturer, species antibody was raised in, antibody catalog and lot # | Dilution used |
| --- | --- | --- | --- |
| Gonadotropin- releasing hormone (GnRH) | GnRH decapeptide (mammalian sequence: pGlu-His-Trp-Ser-Tyr-Gly-Leu-Arg-Pro-Gly-NH_2_) conjugated to bovine thyroglobulin | Mouse anti-GnRH monoclonal, HU11B, Lot # 4. Produced in Dr. Henryk Urbanski’s laboratory, Oregon Primate Center. BALB/c mice were immunized and hybridoma clones were produced [42]. | 1:100 for TEM |
| Gonadotropin- releasing hormone (GnRH) | GnRH decapeptide (mammalian sequence: pGlu-His-Trp-Ser-Tyr-Gly-Leu-Arg-Pro-Gly-NH_2_) conjugated to bovine thyroglobulin | Rabbit anti-GnRH polyclonal antibody, HU60. Produced in Dr. Henryk Urbanski’s laboratory, Oregon Primate Center [35]. | 1:1000 for TEM and confocal |
| Vesicular glutamate transporter-2 (vGluT2) | Recombinant protein from rat vGluT2, derived from protein coded by SLC17A6 solute carrier family 17 (sodium-dependent inorganic phosphate cotransporter), member 6 | Mouse anti-vGluT2 monoclonal antibody, MAB5504. Produced by Millipore-Chemicon (Billerica, MA). | 5 μg/ml for TEM and confocal |
| Vesicular glutamate transporter-1 (vGluT1) | Synthetic peptide from rat vGluT1 protein; no cross-reactivity with vGluT2. | Guinea pig anti-vGluT1 polyclonal antibody, AB5905. Produced by Millipore-Chemicon (Billerica, MA). | 1:1000 or 1:5000 for confocal |
| Estrogen receptor- alpha (ER-alpha) | Direct against the last 14 amino acids of the rat estrogen receptor alpha. | Rabbit anti-ER alpha antibody (C1355). Produced by Upstate Biotechnology (Waltham, MA) | 1:20,000 dilution for confocal |

TEM: transmission electron microscopy
