# Supplementary material for: Expression of Vesicular Glutamate Transporter 2 (vGluT2) on Large Dense-Core Vesicles within GnRH Neuroterminals of Aging Female Rats
Source: PLoS One. 2015 Jun 8;10(6):e0129633. doi: 10.1371/journal.pone.0129633 (PMC4459826; doi:10.1371/journal.pone.0129633)
Supplement: S1 Text — (DOCX) [file pone.0129633.s002.docx]

Using confocal images and physical disector method to quantify the density of GnRH and vGluT2 puncta in the median eminence of the hypothalamus

Imaging software: Image J 1.45s, National Institutes of Health, USA. <http://imagej.nih.gov/ij>

Set region of interest (ROI). Check confocal images and decide region of interest. The pericapillary border in the median eminence is convoluted and the shape varies between animals, therefore we created five 40 μm diameter circles along the pericapillary border to represent ROI within 40 μm of distance to the portal capillary. Add circles to ROI manager. Edit > Selection > Add to manager.


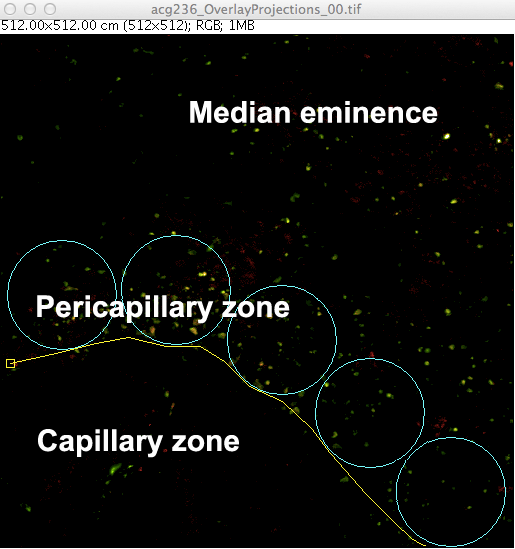


Normalize images: Normalize a pair of confocal images (one reference image and one adjacent image) and make them into binary images.


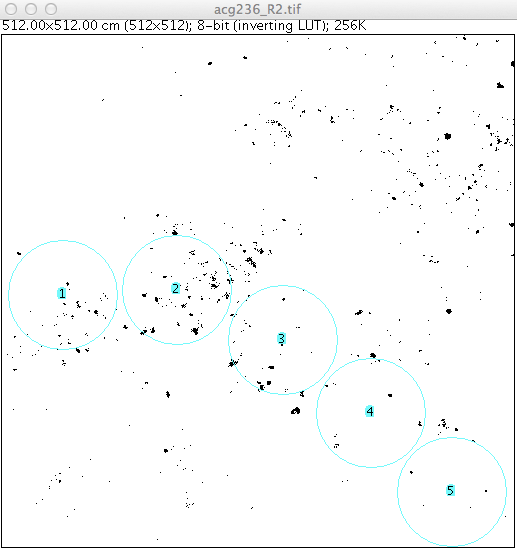


Analyze particles (puncta) in reference image and adjacent image:

Analyze the number of punta in ROI using Analyze > analyze particles.

Analyze particles (puncta) in stacked image:

- Make the paired images into a stack using Image > Stacks > Images to stack
- Find colocalized particles (puncta) using Image > Stacks > Z Project… > Sum slices
- Remove uncolocalized particles by Image > Adjust > Threshold…
- Analyze colocalized particles (puncta) using Analyze > Analyze particles


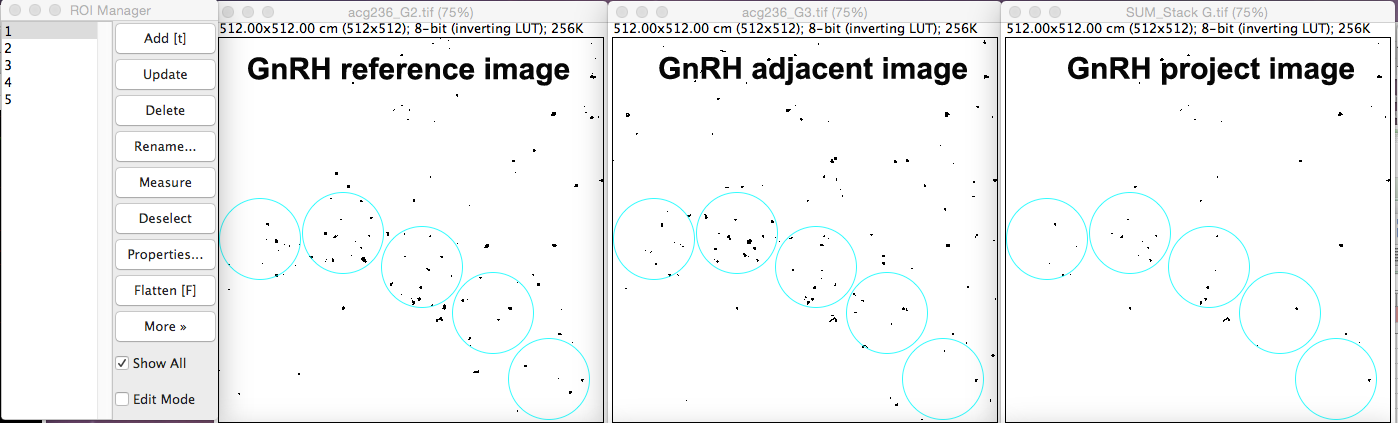


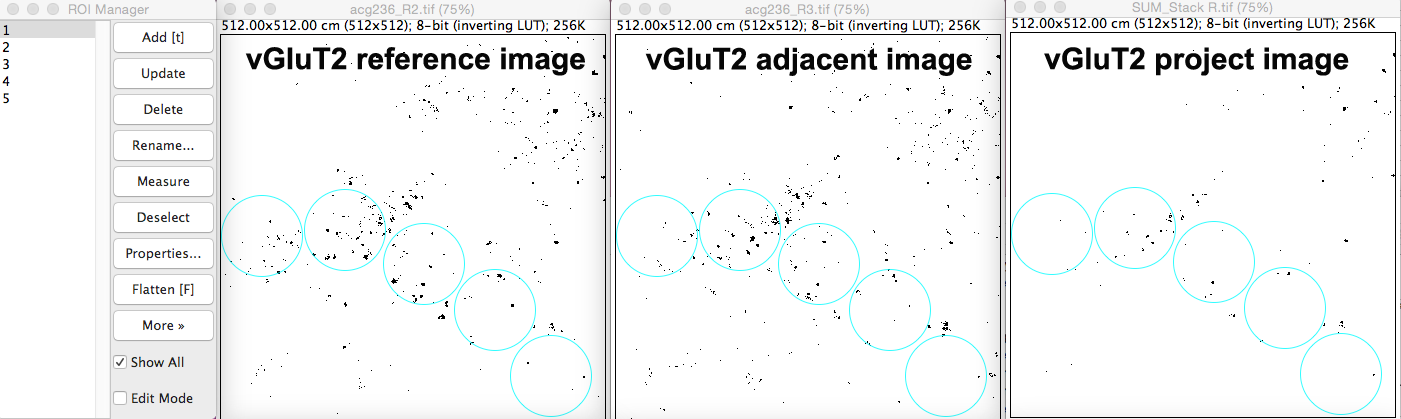


Analyze GnRH and vGluT2 colocalized particles:

GnRH and vGluT2 images in the same scanning plane were stacked, Z-projected and thresholded to create a new image as GnRH-vGluT2 reference image. This reference image was compared to a GnRH-vGluT2 adjacent image, to create a GnRH-vGluT2 project image by Stack and Z-project function as described above.


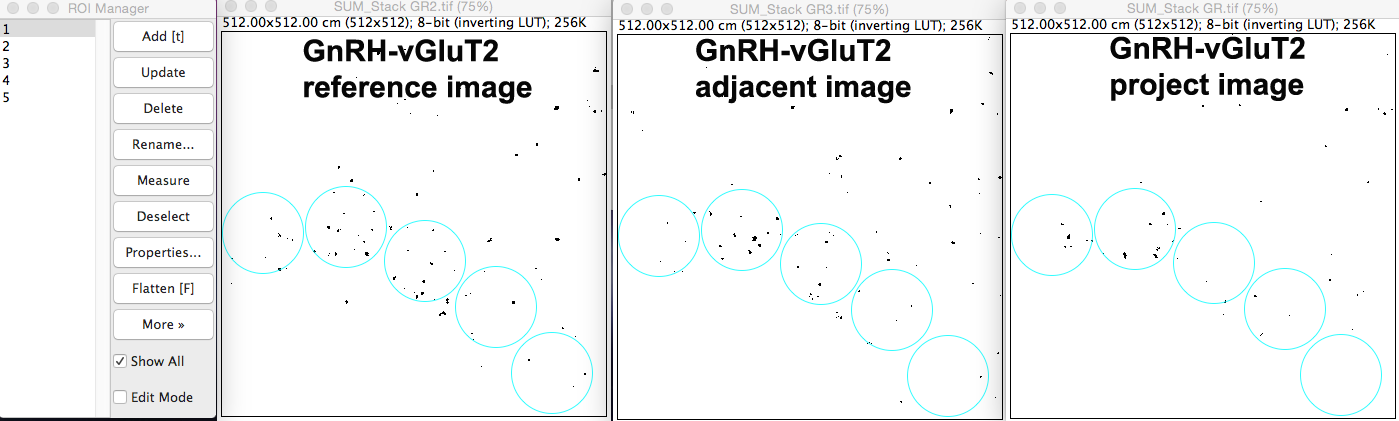


Batch analysis and Macro function: To automatically create stacked and projected images, and analyze particles, we used the Macro record function for batch analysis after manually chosen the pericapillary ROI: Plugins > Macros > Record…

Estimate density of puncta:

The density of GnRH or vGluT2 puncta in the pericapillary area was estimated as:

(# particles in reference image – # particles in project image) / Volume analyzed.

Volume was estimated based on the areas of ROI and the distance between two images.
